# The Collective Investigation Record of the British Medical Association

**Published:** 1884-09

**Authors:** 


### Notes on Books.

---

*The Collective Investigation Record of the British Medical Association.* Vol. II. July, 1884.

PROBABLY no scheme for the advancement of medical science was ever started under more favourable auspices than this collective investigation one of the British Medical Association. Prominent leaders in the profession, praised, and promoted it on platform, paper, and committee in the metropolis; and emissaries of accredited talent and eloquence were sent into the provinces to preach to us this new gospel of scientific research. Everywhere their doctrines have been received with a chorus of assent; questions in scientific medicine were to be answered by the overwhelming mass, and not by the unsupported individual; the untrustworthiness of individual labours in the closet was to be replaced by the generalised experiences of the practitioner in public; and the groping investigations of the medical theorist were to be supplemented, or rather replaced, by the accurate records of the practical worker, as figures, facts, and statistics. *Parturiunt montes, nascetur*—let us see what.

We have before us, in the shape of a goodly octavo of 208 pages, the second volume of records of this Collective Investigation Committee. The greater portion of the volume is concerned with a Report on Acute Pneumonia, the lesser portion with a preliminary report on Puerperal Pyrexia. This lesser portion, as being incomplete, we shall not here deal with, but shall content ourselves with an examination of the results of the major effort. Considering the amount expended on this work—six hundred pounds—we have a right to expect something noteworthy.

We have systematised for us, under various headings, the reports of 1,065 cases. The first thing one naturally turns to is the trustworthiness of the reporters. The men who are most likely to give us satisfactory answers to the queries propounded are the various teachers in our medical schools, the physicians to our hospitals, the men whom, in consultation or general practice, we know to have most experience of the complaint and most knowledge of it. We have gone carefully over the names of those who have made the returns, and we have discovered among them scarcely one which, either from general reputation in these islands or from local reputation in our own district, deserves to be heard as of any authority. That our hospital and consulting physicians practically ignore these inquisitive cards there can be no doubt. The fact is patent to anyone who looks. These are the men who see most of the disease, and know most of the subject, and who are most competent to give returns. Why do they ignore the queries? Do they distrust the process? We fear there is no other explanation possible.

To begin with, therefore, we submit that those most competent to answer refuse to do so, and the trustworthiness of the report is thus, in the first place, weakened.

The report begins with two articles: one on "Epidemics of British Pneumonia," by Dr. Octavius Sturges; the other on "Foreign Pneumonia," by Dr. Sidney Coupland. The nature of these short *résumés* makes us wish that each of these gentlemen had been presented with £300 to give us the best of their brains on the subject. As it is, in their preliminary notes, these two tell us far more than all the other thousand odd do; and there is no saying how much more knowledge the stimulus of a £300 prize to each might have added.

The first table has reference chiefly to the distribution of cases over the seasons. As the summariser judiciously remarks, "An inquiry that deals with but a small proportion of cases occurring in the country is hardly one from which to draw precisely accurate conclusions." We agree with him, and say nothing further than that the greatest number of returns would probably be made not very long after the issue of the cards—which would agree with the table.

As to the "Observers and Localities," the reporter says:—"Concerning the geographical distribution of the disease, no inferences can be drawn from these returns." We agree with him here also, and ignore the appended table accordingly.

Abstracts exhibiting the number of cases and the mortality at various ages, and subsequent on dietetic habits, follow. We are told that the maximum mortality of both sexes under 70 is between 50 and 55 years, and this we are asked to believe from a grand total of 26 males and 15 females! It seems that drunkenness and starvation increase mortality, which is nothing new; but, to the credit side of the account, children are included among the total abstainers and the temperate, giving very impressive figures, certainly, but a mode of compiling statistics which we would expect rather from a temperance hospital than from a committee of our leading medical association.

Abstract IV. actually shews that the worst sanitation, both of houses and districts, gives the lowest mortality! This is so startling that we must quote verbatim:—"If, however, the instances where sanitation is reported 'good' both for house and district be compared with those in which it is 'indifferent' or 'bad' both for house and district, it will be found that Series B shews the worst of the three. Yet this is the series of lowest mortality; and the 126 exhibiting bad sanitary conditions have a mortality of but 16, or 1 in 8; while the 144 under good sanitary conditions have a mortality of 26, or 1 in $5\frac{1}{2}$." What a commentary on the value of barren statistics!

The next abstract refers to the locality and climatic conditions. The veriest tyro in statistical manipulation would know that the tables here presented would be absolutely valueless unless compared at the same time with the actual proportions of population residing on dry or damp soil, exposed or confined situations, the winds prevailing in different parts and at various seasons, and so forth. It is the same as if we had concluded that because only one case was reported from Bristol, with its quarter of a million or so of inhabitants, and two cases occurred on the breezy and barren Mendips, therefore pneumonia was *twice* as prevalent in exposed as in low-lying situations. Seriously, and without exaggeration, this is the line of reasoning followed.

Abstract VII. is simply useless.

Abstract VIII. deals with premonitory symptoms and the onset of the attack. As the writer says, "It would be undesirable to go behind these returns, and to arbitrarily decide what should or should not be considered as a premonitory symptom." Why, then, ask for the figures? And surely it did not want a thousand odd replies from men who have passed their examinations to tell us less than the most ordinary text-book.

Abstracts IX., X., and XI., dealing with the part of the lung affected, the duration of the fever, and the termination of the fever respectively, present us with no new facts. Abstract XII., referring to sequelæ, tells us, among other things of about equal value, that debility followed after 14 cases of acute pneumonia, cough in 12, and bronchitis in 10, out of a total of 1,065 cases. To find cough and bronchitis occupying separate tables as sequelæ of pneumonia is curious enough; but to find debility classed as an independent sequela of one of our most dangerous acute diseases in only 14 cases out of a thousand is simply ridiculous.

The *practical* outcome of the replies of all these practitioners we give in the writer's own words:—"It is manifest that the lines of treatment are so various as not to permit of any value being attached to a comparison of mortality." Surely this is the classical mouse of the mountains in travail at last!

In conclusion, we submit that this is not the way to promote science,—by polling the impressions of its practitioners. The variables and contingencies are so numerous and so important that they cannot, as in this instance, be ignored. To ignore these is to nullify the whole work. And even if the results of this questioning were to be every one trustworthy, drawn up, criticised, and tabulated by skilled observers, we fail to see what new truth this galloping over the surface of disease can bring forth. We have been galloping over disease for centuries, and the outcomes are abundant enough in myriads of works in a score of languages. We want now-a-days to attack disease from the bottom. The work must be one of concentrated labour by the most penetrating intellects, with every opportunity to study disease at the bedside and in the laboratory, and not of vague recollections jotted down in the midst of other pursuits by the rank and file, and huddled into columns by committees. No doubt the Collective Investigation Committee will soon come to a natural and timely end; in the meantime we would express our regret that so much money of the British Medical Association, which might go to its excellent benevolent fund, should be expended on an undertaking so palpably useless as this one.

